# Extracellular vesicles from *Heligmosomoides bakeri* and *Trichuris muris* contain distinct microRNA families and small RNAs that could underpin different functions in the host

**DOI:** 10.1016/j.ijpara.2020.06.002

**Published:** 2020-08

**Authors:** Ruby White, Sujai Kumar, Franklin Wang-Ngai Chow, Elaine Robertson, Kelly S. Hayes, Richard K. Grencis, María A. Duque-Correa, Amy H. Buck

**Affiliations:** a Institute of Immunology & Infection Research, School of Biological Sciences, University of Edinburgh, Edinburgh EH9 3FL, UK; b Lydia Becker Institute of Immunology and Inflammation, Wellcome Trust Centre for Cell Matrix Research and Faculty of Biology, Medicine and Health, University of Manchester, Manchester, UK; c Wellcome Sanger Institute, Wellcome Genome Campus, Hinxton CB10 1SA, UK

**Keywords:** RNA interference, Extracellular RNA, Extracellular vesicle, microRNA, siRNA, Helminth, Gastrointestinal nematode

## Abstract

Extracellular vesicles (EVs) have emerged as a ubiquitous component of helminth excretory-secretory products that can deliver parasite molecules to host cells to elicit immunomodulatory effects. RNAs are one type of cargo molecule that can underpin EV functions, hence there is extensive interest in characterising the RNAs that are present in EVs from different helminth species. Here we outline methods for identifying all of the small RNAs (sRNA) in helminth EVs and address how different methodologies may influence the sRNAs detected. We show that different EV purification methods introduce relatively little variation in the sRNAs that are detected, and that different RNA library preparation methods yielded larger differences. We compared the EV sRNAs in the gastrointestinal nematode *Heligmosomoides bakeri* with those in EVs from the distantly related gastrointestinal nematode *Trichuris muris,* and found that many of the sRNAs in both organisms derive from repetitive elements or intergenic regions. However, only in *H. bakeri* do these RNAs contain a 5′ triphosphate, and Guanine (G) starting nucleotide, consistent with their biogenesis by RNA-dependent RNA polymerases (RdRPs). Distinct microRNA (miRNA) families are carried in EVs from each parasite, with *H. bakeri* EVs specific for miR-71, miR-49, miR-63, miR-259 and miR-240 gene families, and *T. muris* EVs specific for miR-1, miR-1822 and miR-252, and enriched for miR-59, miR-72 and miR-44 families, with the miR-9, miR-10, miR-80 and let-7 families abundant in both. We found a larger proportion of miRNA reads derive from the mouse host in *T. muris* EVs, compared with *H. bakeri* EVs. Our report underscores potential biases in the sRNAs sequenced based on library preparation methods, suggests specific nematode lineages have evolved distinct sRNA synthesis/export pathways, and highlights specific differences in EV miRNAs from *H. bakeri* and *T. muris* that may underpin functional adaptation to their host niches.

## Highlights

• Many small RNAs in extracellular vesicles (EVs) from diverse nematodes derive from intergenic and repetitive elements.
• The parasite microRNA (miRNA) content of EVs is consistent across multiple EV isolation protocols and laboratories.
• *Trichuris muris* and *Heligmosomoides bakeri* EVs contain distinct parasite miRNA family members.
• *Trichuris muris* EVs purified across different laboratories consistently contain more mouse miRNAs than *H. bakeri*.
• Comparisons of helminth EV cargos could help identify sRNAs involved in cross-species communication and niche specificity.

## Graphical abstract

**Figure:**
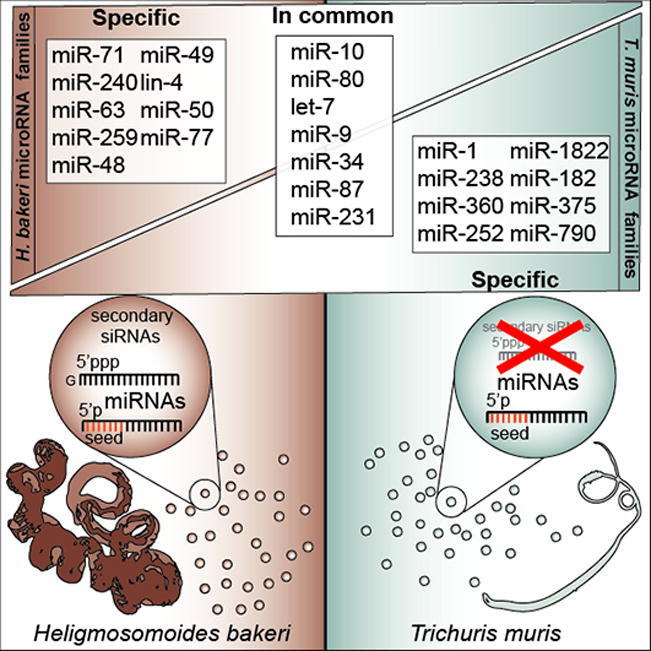

## 1 Introduction

In order to maintain long-term infections, helminths have evolved mechanisms to directly modify the host environment to favour their survival. An emerging mechanism of host modulation by helminths is the release of extracellular vesicles (EVs), which are lipid membrane-enclosed vesicles containing nucleic acid and protein cargo. A growing body of literature demonstrates that diverse parasites have evolved EV cargos that promote parasite survival (Ofir-Birin and Regev-Rudzki, 2019). Many helminth species have been shown to release EVs that can have modulatory actions on host cells *in vitro* and *in vivo* (Coakley et al., 2015, Eichenberger et al., 2018a, Tritten and Geary, 2018)*.* Yet despite the ubiquity of EV release across species, the functions reported for EVs from different helminths are diverse. For example, EVs from *Heligmosomoides bakeri* (also referred to as *Heligmosomoides polygyrus* or *Nematospiroides dubius*) (Behnke and Harris, 2010, Reynolds et al., 2012), have been shown to suppress IL-33 driven Th2 responses, which are integral to worm clearance mechanisms (Buck et al., 2014, Coakley et al., 2017). Similarly, *Echinostoma caproni* EVs induce a mixed Th2 and regulatory immune response that proves partially protective in vaccination models (Trelis et al., 2016). On the other hand, EVs from *Schistosoma japonicum* and *Brugia malayi* parasites polarise macrophages towards an inflammatory M1 phenotype, and in the case of *S. japonicum* this is thought to prevent M2 mediated hepatic fibrosis (Wang et al., 2015, Zamanian et al., 2015, Liu et al., 2019). EVs not only target haemopoietic immune cells, but can also target epithelial cells to regulate their responses. Specifically, EVs from *Opisthorchis viverrini* can enter cholangiocytes, the epithelial cells of the bile duct, and induce their proliferation, potentially contributing to the carcinogenic properties of this liver fluke (Chaiyadet et al., 2015). While the effects of *Trichuris muris* EVs on immunity are not yet fully characterised, their administration confers protection against subsequent infection (Shears et al., 2018). The uptake of *T. muris* EVs has been demonstrated in colonic organoids (Eichenberger et al., 2018b), and more recently in caecal organoids where *T. muris* EVs caused a downregulation in the expression of interferon response genes (Duque-Correa et al., 2020). The immunomodulation achieved by EVs from different helminth species may depend on both the specific EV cargo molecules, and their effects on the particular target cells they encounter during infection.

An intriguing question then is whether, and how, the cargo of the EVs underpin these different functional effects. Our aim here was to compare the cargo of two rodent-infective gastrointestinal nematodes, *H. bakeri* and *T. muris*, that reside in distinct intestinal environments, the small intestine and the caecum, respectively. *Heligmosomoides bakeri* is a clade V nematode, which establishes infection through L3s via the oral route. In the duodenum, larvae invade the stroma and undergo maturation, to re-emerge in the lumen as adult worms (Reynolds et al., 2012). *Trichuris muris* is a distantly related clade I nematode that infects hosts via the oral route as eggs (Coghlan et al., 2019). Upon arrival in the caecum and proximal colon, the eggs hatch, liberating larvae that invade the epithelial cells. In this multi-intracellular niche, larvae moult four times and develop into adults (Klementowicz et al., 2012). Worm expulsion of *H. bakeri* and *T. muris* requires the induction of type 2 immune responses by their host, and in the case of *T. muris* expulsion is dependent on the production of IL-13 specifically (Bancroft et al., 1998). These parasites can both modulate host immunity and counteract the type 2 responses by releasing excretory/secretory (ES) products. Although we have only scratched the surface in understanding the functional properties of ES products, some of the secreted proteins have illuminated key host pathways that are modulated. For example, *H. bakeri* ES products contain a transforming growth factor (TGF)β mimic which skews the immune system towards a regulatory response, allowing worms to persist in their host (Johnston et al., 2017). *Heligmosomoides bakeri* ES products also contain the protein HpARI, which binds and abrogates the effects of IL-33, in turn diminishing type 2 immunity (Osbourn et al., 2017). *Trichuris muris* ES products are dominated by a single protein p43, which binds IL-13 and blocks its function (Bancroft et al., 2019). Many additional parasite-secreted proteins await further characterization, however EVs are also released from these parasites, which could enable coordinated activities of multiple cargo molecules transferred to host cells. Studies illustrate that blocking helminth EVs by vaccination induces partial immunity to infection, suggesting that EVs are functionally important during infection (Coakley et al., 2017, Shears et al., 2018). The detailed understanding of the functional properties of these helminth EVs, and the cargo underpinning these functions, is still lacking.

The EVs from both *T. muris* and *H. bakeri*, as well as those from many other species, contain microRNA (miRNA) cargo (Lefebvre and Lécuyer, 2017). miRNAs are ~22 nucleotide (nt) sequences that mediate gene regulation, and are highly conserved across the animal kingdom (Bartel, 2018). miRNAs are transcribed from genomic loci and form hairpins, which are then processed by RNase III enzymes to produce mature miRNAs. miRNAs are by far the most widely characterised type of small RNAs (sRNAs) described in EVs, however they are not assumed to be the only, or most dominant, sRNA present. For example, mammalian EVs contain fragments of rRNAs, tRNAs and Y RNAs, as well as other classes of non-coding RNAs, and mRNAs (Mateescu et al., 2017). Similarly, many of these classes of sRNA have also been found in EVs and total ES products from helminths including *H. bakeri* (Buck et al., 2014, Chow et al., 2019), *Schistosoma mansoni* (Nowacki et al., 2015), and the filarial nematode *Litosomoides sigmodontis* (Quintana et al., 2019). However, some helminth EVs may also have unique classes of sRNA (compared with mammals) that are specific to their evolutionary lineage. For example, we recently found that a specific type of small interfering (si)RNA dominates the EVs of *H. bakeri* and these siRNAs associate with one specific extracellular Argonaute protein (Chow et al., 2019). The *H. bakeri* siRNAs are classified as “secondary siRNAs” because they are synthesised by RNA-dependent RNA polymerases (RdRPs) and contain a 5’ triphosphate with a preference to start with a Guanine (G). These may be restricted to certain nematode species (Sarkies et al., 2015), and their detection requires that RNA is polyphosphatase-treated prior to library preparation. Here, we aimed to determine and compare the sRNA classes present in EVs from *H. bakeri* and *T. muris* to understand common or distinct features of their sRNA cargos that might enable different functional effects when internalised by host cells and to qualify this in the context of how different isolation procedures might impact results. We found that the differences in EV isolation methods had little effect on the relative miRNA abundances detected for *H. bakeri,* while variations in library preparation kits and polyphosphatase treatment had a bigger impact. We also compared *T. muris* EVs prepared here with previously described and re-analysed datasets of *T. muris* EVs (Tritten et al., 2017, Eichenberger et al., 2018b). We found a good correlation between relative miRNA abundances across the libraries, despite different purification methods and laboratories. When comparing *T. muris* and *H. bakeri* EV sRNAs we discovered that a large portion of EV sRNAs in both *T. muris* and *H. bakeri* derive from intergenic or repetitive elements, although only in *H. bakeri* can the sRNAs from these regions be classified as secondary siRNAs that contain a 5’ triphosphate. We further show that the miRNAs identified in the EVs are highly reproducible across different purification protocols, and we find very distinct miRNA gene families in EVs when comparing the two parasites. Our data suggest that the EV cargoes could elicit distinct functions in regulating host gene networks.

## 2 Materials and methods

### 2.1 *Trichuris muris* EV purification and library preparation

*Trichuris muris* EVs were purified from the ES products of *T. muris* as described previously (Shears et al., 2018), then EVs were isolated by ultracentrifugation (UC), and concentrated using a Vivaspin 6 spin 5 kDa MWCO column. The size and concentration of EVs were assessed by Nanoparticle Tracking Analysis using the NanoSight LM10, and the quality of EVs was assessed by transmission electron microscopy (TEM), which is described in Duque-Correa et al. (2020). RNA was extracted using the miRNeasy mini kit (Qiagen). For samples that were DNase treated, these were incubated for 10 min at room temperature (RT) with RNase-free DNase I with additional RNasin RNase inhibitor (Promega). Polyphosphatase treatment was performed using RNA 5′ polyphosphatase (Epicentre) following the manufacturer’s instructions. RNA that was enzymatically treated, either with DNase or 5′ polyphosphatase, was subsequently purified by ethanol precipitation. Libraries were prepared using the CleanTag small RNA Library prep kit (TriLink) following the manufacturer’s instructions. Adapters were diluted 1:12 and 18 PCR cycles were used.

### 2.2 *Heligmosomoides bakeri* EV purification by fractionation and library preparation

*Heligmosomoides bakeri* ES products were harvested as described previously (Chow et al., 2019). The filtered *H. bakeri* ES products were concentrated in a Vivaspin 20 MWCO 3 kDa concentrator (Sartorius, Göttingen, Germany). Four hundred μL of *H. bakeri* ES products (concentration 5 mg/mL) were separated into 1 ml fractions by size exclusion chromatography using a Superdex 200 10/300 GL column (GE Healthcare) in PBS with an AKTA basic FPLC system (GE Healthcare). Libraries were prepared using the CleanTag small RNA Library prep kit (Trilink) following the manufacturer’s instructions. Adapters were diluted 1:12 and 18 PCR cycles were used. Quantification and size analysis of *H. bakeri* EVs were performed using the qNano Gold platform (Izon Science). EVs were measured in PBS as electrolyte without dilutions and compared to calibration particles CPC100 (Izon Science). Samples from fractions 1 to 4 were measured using the nanopore NP150 (Izon Science) at 47.2 mm stretch, with voltage 0.6 V and pressure 20 mbar. Data analysis was carried out using the Izon Control Suite software v3.2.2.251 (Izon Science). The EVs from fraction 3 were fixed in 2% paraformaldehyde (PFA), deposited on Formavar-carbon-coated EM grids and treated with glutaraldehyde before treatment with uranyl oxalate and methyl cellulose as described previously (Buck et al., 2014), then visualised in a Philips CM120 TEM (Edinburgh). Images were taken on a Gatan Orius CCD camera.

### 2.3 Sequence analysis

The unprocessed sequence data for each sample were analysed by FastQC (v0.11.8) (http://www.bioinformatics.babraham.ac.uk/projects/fastqc/) to obtain an overview of the sequence data quality. Subsequently, the 3′ sRNA adapter was removed using cutadapt (v2.7) (Martin, 2011), searching for at least a six base match to the adapter sequence. For analysis of sRNAs, only sequences that contained the adapter, were >/=18 nt in length, and did not contain any Ns (uncalled bases) were retained for further analysis. Genome alignments to the *Mus musculus* (version GRCm38, with additional RefSeq rRNA, as all mouse rRNA genes are not present in the genome), *H. bakeri* (GCA_900096555.1), and *T. muris* (GCA_000612645.2) genomes were performed using bowtie (v1.2.2) (Langmead et al., 2009), requiring perfect matches along the full length of the sequence. Combining the data from the alignments, we defined *H. bakeri- or T. muris*-specific sequences as those sequences that matched perfectly and unambiguously to the respective genomes but not to *M. musculus*.

### 2.4 miRNA predictions

miRNAs were predicted using reads that mapped to *H. bakeri* or *T. muris* genomes (these could also map to mouse). We used miRDeep2 v2.0.0.8 (32) with mature and hairpin sequences from miRBase v22 (33) and additional nematode miRNA sequences from *Brugia pahangi* (Winter et al., 2012). miRDeep2 initially predicted a total of 428 miRNAs for *H. bakeri* based on all libraries including life stages reported in Buck et al. (2014), but we kept only 94 that were present at greater than 100 reads in total when summing reads from all *H. bakeri* EV samples, and did not have low complexity sequences. For *T. muris*, 110 of 225 initially predicted miRNAs were kept for the same reasons. We note that one limitation of this approach is that miRNAs that are present at very low copy numbers in only one sample could be excluded. miRNA names were assigned using known miRBase names in a hierarchical fashion, with highest priority assigned to precursors that matched known nematode miRNA precursors. If these did not exist, we named the miRNAs according to exact seed matches in mature sequences to known nematode mature miRNAs. miRNA predictions without a mature seed match to a known nematode mature miRNA were labelled ‘novel’ and these were examined manually and discarded if their structure and read mapping profile did not fit expected miRNA criteria. To assign miRNA family names, we used seed matches to known Nematoda and non-Nematoda miRNA.

### 2.5 Parasite RNA biotypes

Read biotypes (miRNA, rRNA, protein-coding exon, etc.) present in each sample were identified by mapping the short reads to annotated versions of the *H. bakeri* and *T. muris* genomes. For *H. bakeri*, we used genome version PRJEB15396 nHp.2.0, (downloaded from https://parasite.wormbase.org/Heligmosomoides_polygyrus_prjeb15396) and the annotations as in Chow et al. (2019) downloaded from WormBase ParaSite (Howe et al., 2017). For *T. muris*, the genome version used was PRJEB126 TMUE3.0 (downloaded from (https://parasite.wormbase.org/Trichuris_muris_prjeb126) (Howe et al., 2017) and it was reannotated in a similar manner to *H. bakeri* as follows: miRNAs were identified using miRDeep2 as above (Friedländer et al., 2012); rRNAs using cmsearch from the infernal (v1.1.2) (Nawrocki and Eddy, 2013) suite to search for eukaryotic rRNAs using covariance models from Rfam (version 14.1)] (Kalvari et al., 2017) and default settings; tRNAs using tRNA-scan-SE (v1.3.1) (Chan and Lowe, 2019); protein-coding exons and protein-coding introns were extracted from the publicly available GFF3 annotation on WormBase ParaSite genome PREJEB126. Repetitive elements were predicted using RepeatModeler v1.0.11 on the draft genome followed by RepeatMasker v4.1.0 (http://www.repeatmasker.org) using RepBase v20181026 Nematoda sequences (Bao et al., 2015).

Short reads between 18 and 30 bases long can map to multiple locations in the genome, but rather than assign them randomly to each possible location, we used ShortStack (v3.8.5) (Axtell, 2013) to assign reads in a way that takes into account the number of unique reads mapping to those locations. As in the *H. bakeri* annotation (Chow et al., 2019), overlapping annotations were assigned in order of priority from miRNAs to introns, so that each position on the *T. muris* genome had only one biotype annotation. The read mappings were intersected with the *H. bakeri* and *T. muris* genome annotations to count the number of reads belonging to each biotype.

### 2.6 Data accessibility

The data discussed in this publication have been deposited in the NCBI Gene Expression Omnibus (Edgar et al., 2002) and are accessible through GEO Series accession number GSE152591.

## 3 Results

### 3.1 Genome mapping of sRNAs in EVs from *H. bakeri* and *T. muris*

**Fig. 1:**
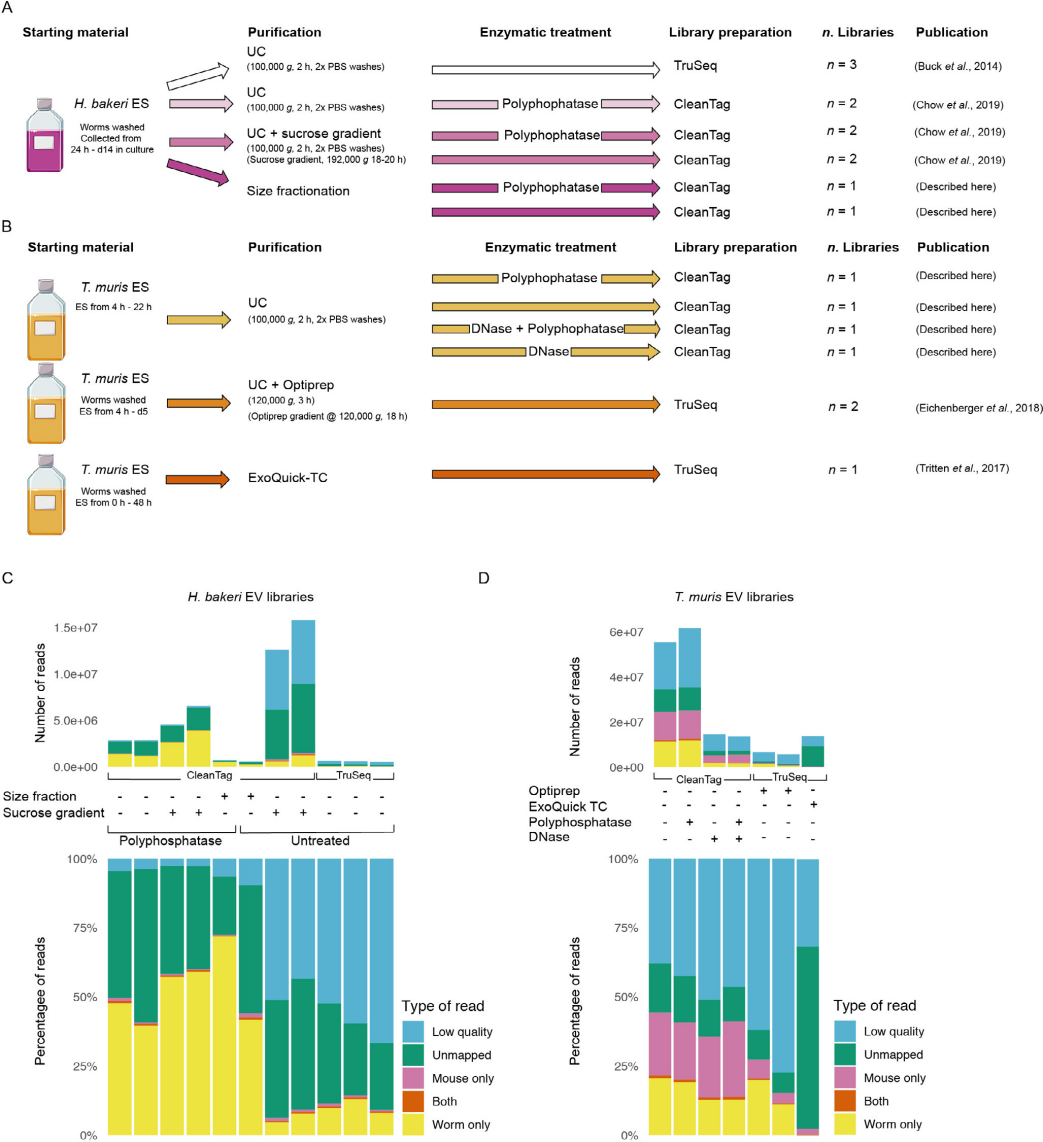
Genome mapping of *Heligmosomoides bakeri* and *Trichuris muris* extracellular vesicle (EV) small RNA libraries. A schematic depicting methods for generation of (A) *H. bakeri* EV small RNA libraries or (B) *T. muris* small RNA EV libraries. Libraries with the same colour arrows were prepared at the same time. For publicly available libraries the publication is cited, and should be referred to for more detailed methodologies. (C–F) The number and proportion of reads that were low quality, did not map to either the mouse or parasite genome (unmapped), mapped equally well to both genomes (both), mapped only to mouse or the respective parasite genomes, are shown for (C) *H. bakeri* small RNA libraries and (D) *T. muris* small RNA libraries. All *H. bakeri* EVs were first purified by ultracentrifugation (UC), with the exception of size fractionation; any further purification steps, sucrose gradient, polyphosphatase treatment, and library preparation protocol (Clean Tag, or TruSeq) are indicated. *Trichuris muris* EVs were all purified by UC except for those purified by Optiprep or ExoQuick as indicated. Treatment with either polyphosphatase or DNase, and the library kit used, are indicated. ES, Excretory/Secretory products; d, day; h, hour.

In order to accurately perform comparisons between EVs from different species, we first sought to understand the extent of variation in sRNAs detected across different isolation procedures in one species. We compared nine publicly available datasets of *H. bakeri* EV libraries generated by our laboratory, with two previously unpublished datasets (Fig. 1A and Supplementary Table S1) and determined the extent to which different isolation methods influenced the sRNAs detected. All quality control of EV purifications can be found in the relevant references, or for EVs and libraries generated and not yet published, the information is provided in Supplementary Fig. S1 of this publication. *Heligmosomoides bakeri* ES products were collected from adult worms from days 2 to 8 (media were replaced on day 4) post-harvest and pooled. ES products from the first 24 h is not included to minimise contamination from host material (Johnston et al., 2015). We compared RNA libraries prepared from EVs isolated by UC, with or without further sucrose gradient purification, or by size exclusion fractionation alone (Fig. 1A). Across the samples we found 5–72% of raw reads mapping to the *H. bakeri* genome and a low percentage of reads mapping to mouse (<2%), or ambiguous reads (<1%) defined as mapping equally well to *H. bakeri* and mouse (Fig. 1D). The high percentage of unmapped reads is not related to adapter fragments in the libraries, but is consistent with extracellular RNA libraries across a range of biological systems and likely reflects background (Heintz-Buschart et al., 2018). To compare the sRNA cargo with *T. muris* EVs, libraries were generated using UC purification of ES products collected between 4–22 h of culture as previously described (Shears et al., 2018). We also re-analysed published sRNA libraries from *T. muris* EVs generated by Eichenberger et al., 2018a, Eichenberger et al., 2018b using Optiprep gradient purification from ES products collected daily from 4 h −5 days of culture, and generated by Tritten and Geary (2018) using ES products from 18-48 h of culture and purified by ExoQuick-TC (Fig. 1B). A detailed schematic of the analysis pipeline for both species is provided in Supplementary Fig. S2. Across the *T. muris* libraries, we find that 0.2–20% of the raw reads mapped to the *T. muris* genome, and 0.2–22% of raw reads mapped to mouse (Fig. 1F). Within *T. muris* libraries there are consistently higher proportions of mouse reads than in *H. bakeri* libraries, irrespective of which laboratories generated these. These differences could be attributed to the harvesting, the number of wash steps prior to culture, and the fact that the first 24 h of culture of ES products from *H. bakeri* is not included (whereas only the first 4 h of culture is excluded in *T. muris* ES products). Increases in the mouse sequences may also relate to the *T. muris* intracellular life stage, which might internalise more host material that is then released in EVs.

### 3.2 EVs from both *H. bakeri* and *T. muris* have a high proportion of sRNAs from repetitive or intergenic regions, however only *H. bakeri* sRNAs contain a 5′ triphosphate

**Fig. 2:**
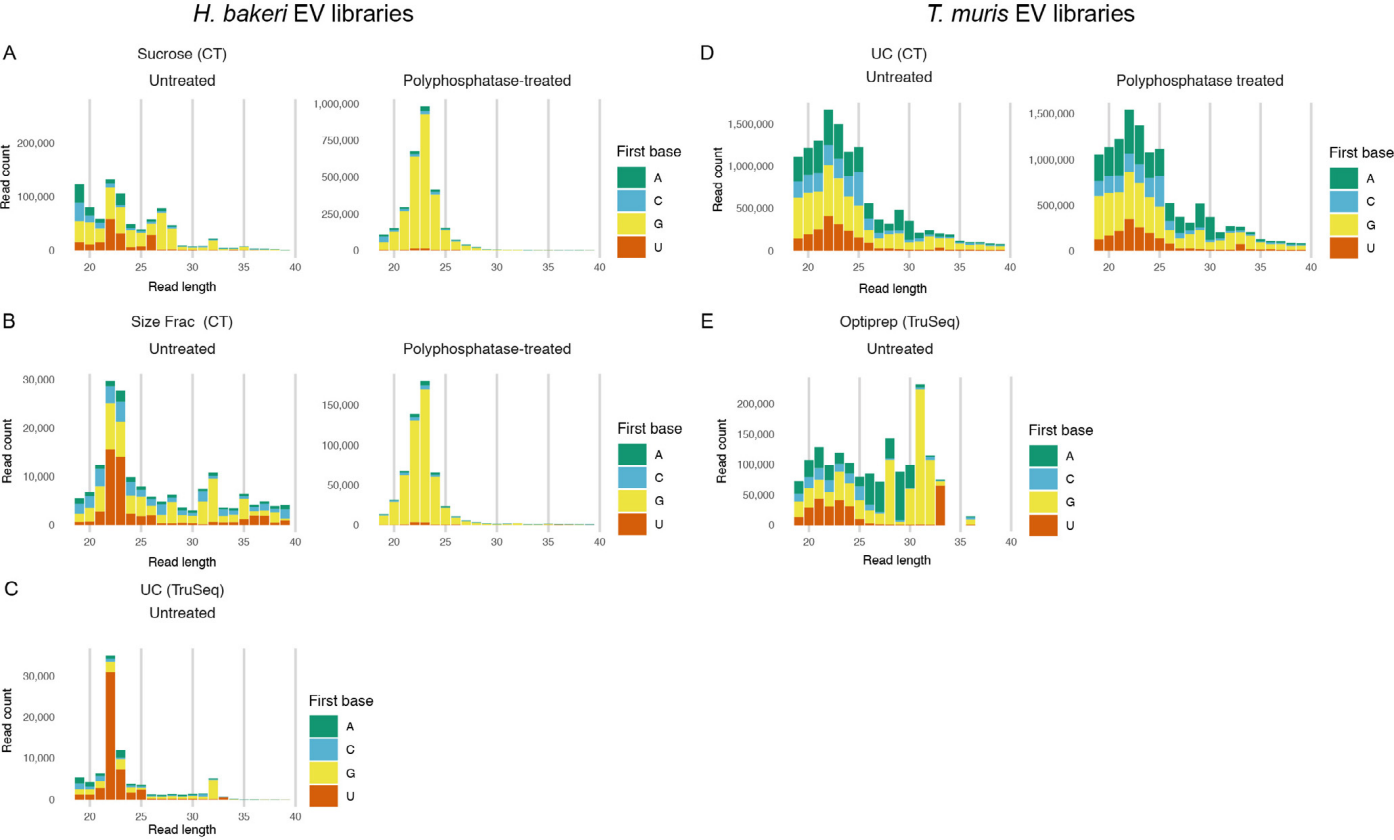
First nucleotide and length distribution for *Heligmosomoides bakeri* and *Trichuris muris* extracellular vesicle (EV) small RNA libraries. Nucleotide length distribution plots of small RNA reads indicating whether the first base of the read is A, C, G or U. (A–C) *Heligmosomoides bakeri* EV libraries and (D and E) *T. muris* EV libraries. (A) EVs purified by ultracentrifugation (UC) + sucrose gradient purification, and prepared using the Clean Tag method, that were either untreated (left) or polyphosphatase-treated (right). (B) EVs purified by size fractionation, and prepared using the Clean Tag method, that were either untreated (left) or polyphosphatase-treated (right). (C) EVs purified by UC and libraries prepared using TrueSeq. (D) EVs purified using UC and libraries prepared with the Clean Tag method that were either untreated (left) or polyphosphatase-treated (right). (E) EVs purified by Optiprep and libraries prepared by TruSeq. Frac, Fractionation; CT, CleanTag.

**Fig. 3:**
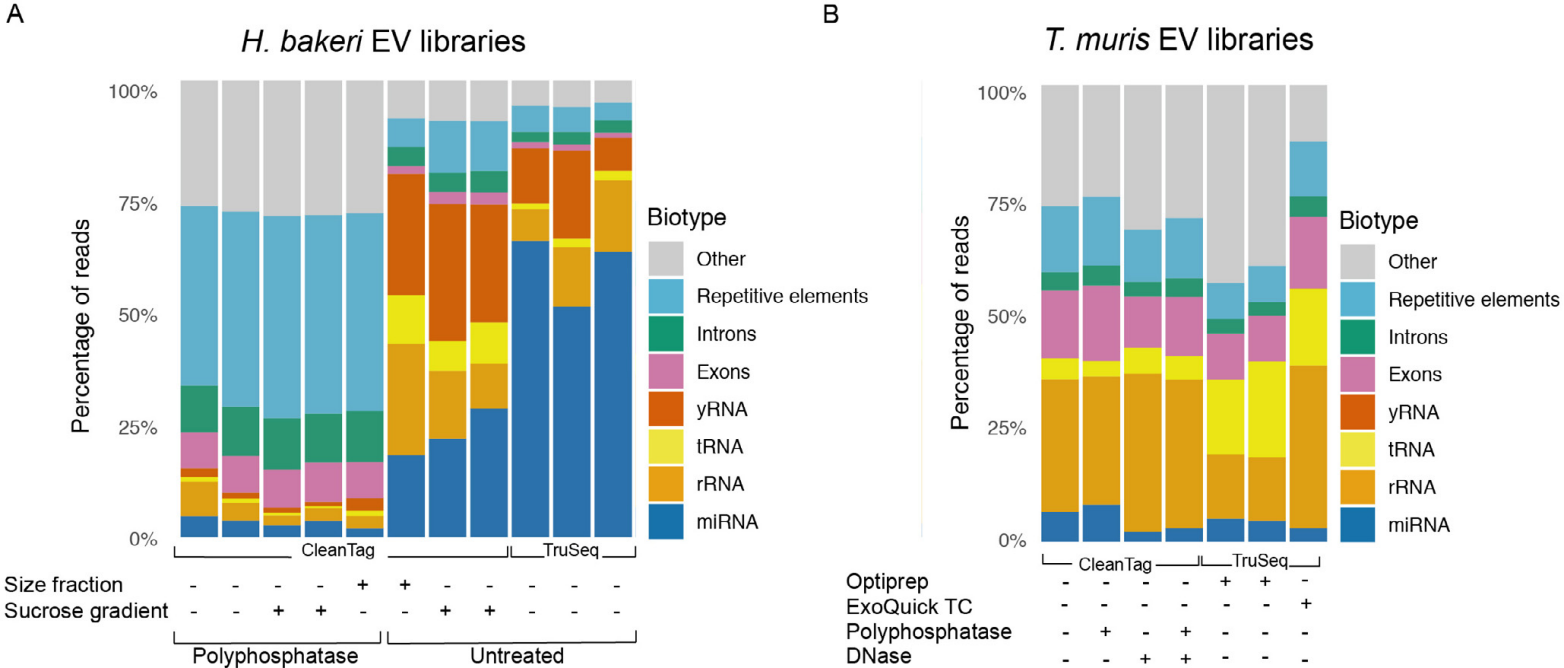
Parasite RNA biotypes for *Heligmosomoides bakeri* and *Trichuris muris* extracellular vesicle (EV) small RNA libraries. The proportion of reads for parasite small RNA biotypes for (A) *H. baker*i EV small RNA libraries or (B) *T. muris* EV small RNA libraries. (A) All *H. bakeri* EVs were first purified by ultracentrifugation (UC); any further purification steps (size fraction, sucrose gradient), polyphosphatase treatment, and library preparation protocol (Clean Tag or TruSeq) are indicated in the figure. (B) *Trichuris muris* EVs were all purified by UC except for those purified by Optiprep or ExoQuick as indicated. Treatment with either polyphosphatase or DNase, and the library kit used, is indicated in the figure. Reads mapping both sense and antisense to biotype categories are included.

To account for all classes of parasite sRNAs in EVs, we made libraries with and without polyphosphatase treatment. Treatment with the 5′ RNA polyphosphatase enzyme dephosphorylates the 5′ triphosphate group to a 5′ monophosphate, allowing the 5′ sequencing adapter to ligate to the RNA. As expected, polyphosphatase treatment of sRNA from *H. bakeri* EVs showed that the dominant category of sRNA is not miRNAs, but rather secondary siRNAs which are ~22–23 nt and begin with G (Chow et al., 2019) (Fig. 2A, B). In contrast, we did not observe differences in the size peak of reads, nor the starting nucleotide in polyphosphatase treated libraries from the *T. muris* EVs, suggesting that the same type of secondary siRNAs identified in *H. bakeri* EVs are not present in *T. muris* EVs (Fig. 2D). This is consistent with the finding that specific RdRPs involved in secondary siRNA biogenesis are not present outside clade III-V nematodes (Sarkies et al., 2015). We suspect that this class of secondary siRNA would also be absent within the *T. muris* adult worms, although this requires experimental confirmation. For *H. bakeri* libraries, the regions in the genome to which the sRNAs map changed dramatically with the polyphosphatase treatment, with an increase in those mapping to repetitive regions or “other” including intergenic regions (Fig. 3A). This is consistent with the secreted secondary siRNAs being derived from these regions of the genome, as described previously (Chow et al., 2019). For *T. muris* EV libraries, a large proportion of reads derived from repetitive or intergenic regions, however there was no change in the proportion of any biotype when polyphosphatase-treated. This suggests that no dominant species of sRNA in EVs from *T. muris* contain 5’ triphosphate moieties (Fig. 3B). *Trichuris muris* EV libraries also contained a large proportion of rRNA fragments, which may be degradation products given their range in size (Supplementary Fig. S3). It is notable that, in contrast to rRNAs, the sRNAs derived from intergenic or repetitive regions tend to show a narrower size distribution between 21 and 25 nt in length (Supplementary Fig. S3).

### 3.3 Robust detection of miRNAs in *H. bakeri* EVs despite varying methodologies for isolation

**Fig. 4:**
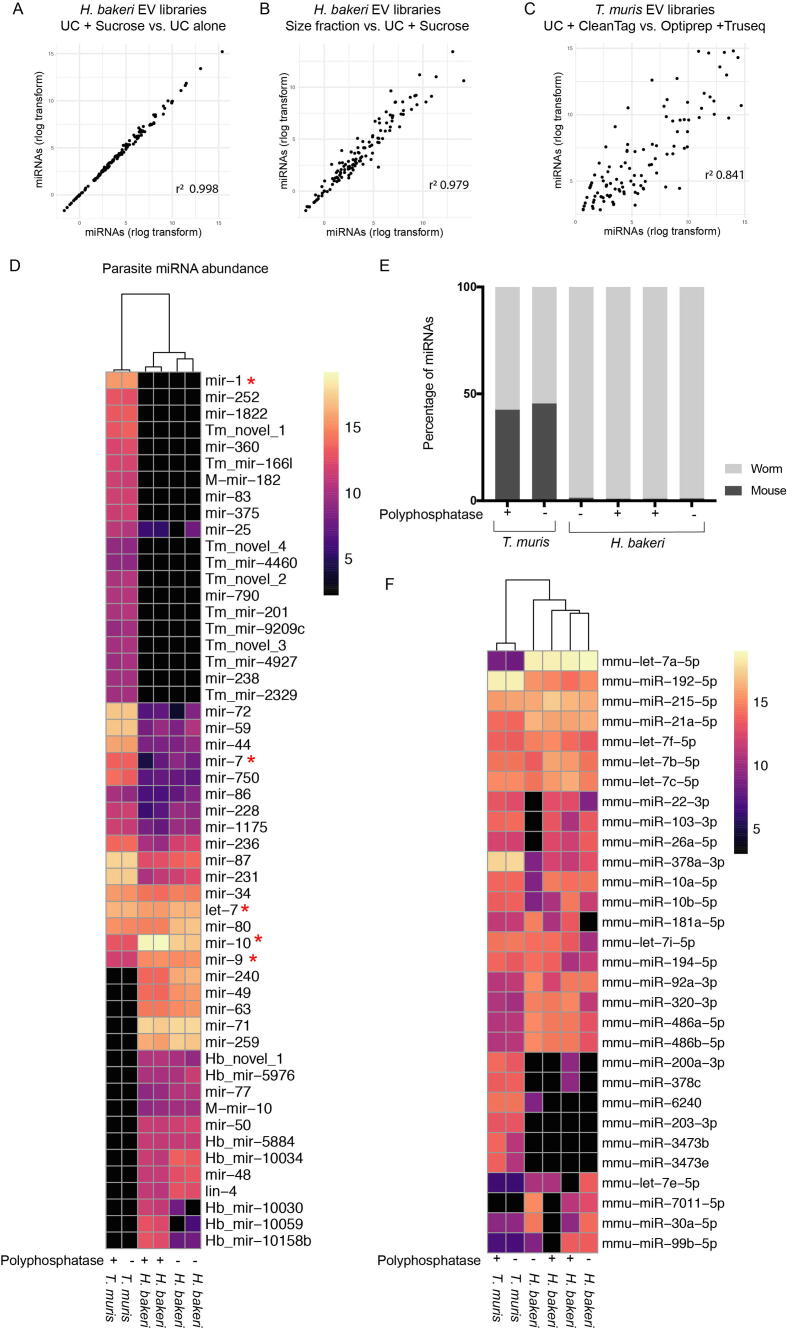
Comparison of microRNA (miRNA) cargo in *Heligmosomoides bakeri* and *Trichuris muris* extracellular vesicle (EV) small RNA libraries. Scatter plots of (A and B) rlog counts for 94 miRNAs, which were present in all of the *H. bakeri* EV libraries plotted and (C) rlog counts for 110 miRNA sequences present in all *T. muris* EV libraries plotted. (A) *Heligmosomoides. bakeri* EV libraries purified by ultracentrifugation (UC) + sucrose gradient versus *H. bakeri* EV libraries purified by UC alone. (B) *Heligmosomoides bakeri* EV libraries purified by size fractionation versus *H. bakeri* EV libraries purified by UC + sucrose gradient. (C) *Trichuris muris* EV libraries purified by UC and prepared using the Clean Tag kit in our laboratory versus *T. muris* EV libraries from Eichenberger et al., 2018a, Eichenberger et al., 2018b, purified by Optiprep and prepared using TruSeq. (D) Heatmap of parasite miRNA abundance broken down by miRNA families in *H. bakeri* and *T. muris* EV libraries (normalised to total parasite miRNA counts in each library). Red asterisks refer to miRNAs that are identical in sequence to mouse miRNAs. MiRNAs which are not identicial to mouse miRNAs, but share a common seed with a mouse miRNA, and not any other nematode miRNA are named with “M-”. MiRNAs which are species-specific are named with “Hb or Tm” at the start. (E) Percentage of identified miRNAs that were from the parasite or from mouse in different libraries. (F) Heatmap of mouse miRNAs found in *H. bakeri* and *T. muris* EV libraries (normalised to total mouse miRNA counts in each library).

The miRNAs represent 2–65% of the parasite sRNA reads in *H. bakeri* EVs, while in *T. muris* the miRNAs make up 2–8% (Fig. 3A, B). However, miRNAs are a class of interest because there is extensive focus on their role in cell-to-cell communication in mammals, and a framework for their capacity to suppress genes through RNA interference (Mateescu et al., 2017). To understand the reliability of parasite miRNAs identified across datasets, we compared the miRNA composition of 11 samples of *H. bakeri* EVs derived from different purification protocols, library preparation methods, and in the absence or presence of polyphosphatase treatment. A total of 94 parasite miRNAs were used for the analysis, based on the requirement that these were present in > 100 reads when summing across datasets (Supplementary Table S2). Principle component analysis (PCA) of the 94 miRNAs present in 11 *H. bakeri* samples indicates that the largest proportion of variance is attributed to the different library preparation kits used, which should be considered for future comparative studies (Supplementary Fig. S4). Interestingly, libraries clustered closely regardless of whether EVs were purified by UC alone, or with the addition of sucrose gradient purification (Supplementary Fig. S4). In particular, sucrose gradient purification did not make a difference in the identity and relative abundance of miRNAs detected compared with UC alone (*r*^2^ = 0.998) (Fig. 4A). This suggests that the *H. bakeri* EVs purified by UC (washed twice with PBS) do not contain non-vesicular miRNA contaminants, or that any contaminants will not be further removed by flotation on a sucrose gradient. Libraries purified by size fractionation clustered further away by PCA (Supplementary Fig. S4), but still show very high correlations in terms of the miRNA cargoes with libraries of EVs purified by UC (*r*^2^ = 0.979) (Fig. 4B).

Next, we compared the miRNA cargo of *T. muris* EVs prepared here, with the cargo from EVs described in Eichenberger et al., 2018a, Eichenberger et al., 2018b, using 110 parasite miRNAs that could be detected across all the samples with high confidence (>100 reads). We saw a strong correlation (*r*^2^ = 0.841) in the parasite miRNA cargo, which is striking considering that the samples were generated in different laboratories, purified by different methodologies, and prepared with different library protocols (Fig. 4C). In particular, there was an overlap of 20 out of the top 25 miRNA families from both sets of *T. muris* EV libraries (Supplementary Table S3). Together, this work suggests that the detection of miRNAs in EVs from a given helminth is robust across purification methods, but also points to potential variation in other classes of sRNAs. For example, the samples from Eichenberger et al., 2018a, Eichenberger et al., 2018b contain a higher proportion of tRNA fragments (Fig. 3B) which are distinguishable as a peak at ~31 nt (Fig. 2E). tRNA fragments were captured in our *T. muris* libraries (Fig. 3B) but in a much lower proportion of total reads, and we detected larger proportions of rRNA fragments.

### 3.4 Differences in parasite miRNA cargo between *H. bakeri* and *T. muris* EVs

To compare the individual parasite-derived miRNAs in *H. bakeri* and *T. muris* EVs*,* we focused on the libraries prepared with the CleanTag library kit in our laboratory, using both untreated and polyphosphatase-treated RNA. First, we assigned miRNA families based on identical seed matches to known Nematoda miRNAs (in cases where there is more than one gene family for a given seed, we have included this information in Supplementary Tables S4, S5). In several cases, the seed sites of the parasite miRNAs did not match a known nematode miRNA, but did match a mouse miRNA. We do not assume these miRNAs share common ancestry between nematodes and mammals but believe these are worth highlighting since the parasite miRNA could potentially mimic the host miRNA function in the host cell. We have classified the gene families of these parasite miRNAs as “M-mir-X” (“M” stands for mouse) (Fig. 4D). miRNAs that did not have a seed match to known Nematoda, or any mammalian miRNA families, were labelled “Hb” or “Tm”, followed by the miRNA name assigned by miRDeep2, or were labelled “novel” if no name was assigned. We then compared the abundance of the top 40 miRNAs from each parasite, combining miRNAs that shared the same seed as one family (Fig. 4D). As expected, samples from the same nematode species clustered together, although some miRNA families are present and highly abundant in both *H. bakeri* and *T. muris* EVs, including: miR-10, miR-9, miR-87, let-7, miR-80, and miR-34 (Fig. 4D). This is consistent with a recent review from Sotillo et al., (2020), which compared miRNA cargo across a large number of helminths, and found miR-10 and let-7 to be common to all species, with miR-71 and lin-4 also common to all nematodes except *T. muris* (Sotillo et al., 2020). In agreement with these findings, we detected miR-71 and lin-4 only in *H. bakeri* libraries. A subset of nematode miRNA families were present in both datasets, but enriched in *T. muris* EVs including miR-44, miR-72, miR-59, and to a lesser extent miR-7 and miR-750. Strikingly, *H. bakeri* and *T. muris* EVs contained many miRNA families that were not detected in the other species: 17 in *H. bakeri* and 19 in *T. muris* of the miRNAs in the top 40 most abundant miRNAs (Fig. 4D and Supplementary Tables S4, S5). The top unique families for *H. bakeri* were miR-71, miR-240, miR-49, miR-259 and miR-63 and for *T. muris* miR-1822 (which has a seed site identical to the host miR-22), miR-252, miR-360 and miR-1. It is worth noting that the miR-1 family, as well as let-7, miR-10, miR-7 and miR-9 families, contain members that are identical in sequence to the host miRNAs (indicated by red asterisks, Fig. 4D) and some of these could in fact derive from the host (detailed further below). Additionally, some species-specific miRNAs were found in the top 40 list that do not have a seed matching to a known miRNA family (Fig. 4D). Overall, the miRNA cargo of EVs from these different nematode species was highly distinct, suggesting they could have different sets of target genes.

### 3.5 Host miRNA content of *T. muris* and *H. bakeri* EVs

Most of the studies on helminth EVs focus only on parasite miRNAs, yet one report in *T. muris* previously suggested host miRNAs can also be present (Tritten and Geary, 2018). In order to examine the host miRNAs in our datasets, we analysed all of the reads mapping to the mouse genome (that do not map to the parasite genomes also) and classified the biotypes present. Most of the mouse RNAs are rRNAs, and to a lesser extent miRNAs and tRNA fragments (Supplementary Fig. S5). To classify the host miRNAs present in *H. bakeri* and *T. muris* samples, we combined all libraries and quantified them using miRDeep2, requiring > /=100 read counts for inclusion, when summing across the samples. PCA showed a clear separation of EVs from *H. bakeri* and *T. muris*, irrespective of which laboratory isolated these or how the libraries were prepared (Supplementary Fig. S6). We found only trace quantities of the host miRNAs in *H. bakeri* libraries (Fig. 4E). In contrast, in the *T. muris* libraries the host miRNAs are nearly as abundant as those from the parasite (Fig. 4E). We then compared the relative abundance of individual host miRNA sequences in *H. bakeri* and *T. muris* EV libraries (as was done for the parasite miRNAs). As expected, each of these samples clustered by nematode (Fig. 4F). Although some host miRNAs were dominant in both (10 of the top 20 most abundant miRNAs were common between the parasite EVs) a large number of miRNAs present in *T. muris* libraries were not detected in those of *H. bakeri,* for example miR-378 family members (Fig. 4F, Supplementary Table S6). These miRNAs are not similar to any of the *T. muris* miRNAs, and therefore are not expected to result from sequencing errors of parasite miRNAs. Most of the host miRNAs identified here also do not match the parasite genomes (even when allowing up to two mismatches, Supplementary Table S7) and are present in distinct abundance rankings between species, suggesting these are not laboratory contaminants or unclassified parasite RNAs (Fig. 4F). One host miRNA was significantly more dominant in *H. bakeri* compared with *T. muris* samples and this miRNA (let-7a-5p) is identical in mouse and nematode. Since the host miRNAs are so low in abundance in *H. bakeri,* it is likely that these let-7a sequences are in fact derived from the parasite but contain 3′ end modifications that cause them to map perfectly to mouse but not the parasite. The fact that *T. muris* EV libraries contain a larger dominance of host miRNAs compared with *H. bakeri* EVs could be attributed to the intracellular life stage of *T. muris,* their close contact with epithelia throughout infection, and differences in washing worms post-harvest. Our comparison also demonstrates that the miRNA populations in the EVs from these two parasites are quite distinct. These differences may relate to difference in miRNA content in the caecal versus small intestine niches of the parasites or differences in host miRNAs that are up-regulated during each infection.

## 4 Discussion

Purification methods for helminth EVs vary between research groups and there is extensive interest now in documenting whether and how these variables can impact the properties of EVs and the cargo molecules that are identified, as outlined for mammalian EV research (Théry et al., 2018). Our results suggest that detection of the EV miRNA cargo is relatively consistent across different purification strategies for *H. bakeri* EVs, for example adding a sucrose flotation step after UC purification (that was carried out with two PBS washes) does not yield any difference in the miRNA detected compared. We also show a strong correlation in the EV miRNA cargo sequenced by different laboratories for *T. muris*, even when different purification methods (UC versus Optiprep gradient purification) and library preparation methods (CleanTag versus TruSeq) were used. Our comparisons also yield the result that host miRNAs are in much greater abundance in *T. muris* EVs compared with *H. bakeri* EVs, and this was consistent across libraries from different research groups. This may have functional implications if the host miRNAs are also transported into specific cells during infection. At present it is not known if the host miRNAs are truly within the EVs or if these co-purify, but it is notable that they are consistently detected when EVs are purified by either UC or Optiprep gradients. One variable which may introduce the largest variation in the sRNAs recovered after sequencing is the library preparation kit used, since biases can be introduced in relation to the ligation efficiency of adapters to different sRNAs (Fuchs et al., 2015). Also, the size selection of the libraries dictates the other classes of sRNA that will be detected. A notable example here is the proportion of tRNA fragments detected, which are generally ~31–33 nt in length and are ubiquitous in extracellular environments (Tosar et al., 2018, Watson et al., 2019). The tRNA fragments were present in a much higher proportion of samples purified by Eichenberger et al., 2018a, Eichenberger et al., 2018b which either represents a biological difference of the EV RNA cargo identified in their study (using Optiprep gradient purification) or a technical difference based on biases in the library method or differences in how libraries were size selected. The previous study also identified mRNA transcripts in *T. muris* EVs which strikingly were dominated by reverse-transcriptase-like protein families (Eichenberger et al., 2018b).

One thing that is clear from our analysis is that a large proportion of parasite sRNA reads derive from intergenic or repetitive regions of the genome. Together, these make up >75% of the reads in the polyphosphatase-treated *H. bakeri* EV libraries, and ~24–51% across *T. muris* EV libraries, irrespective of the purification method or laboratory generating the libraries. These regions of the parasite genomes are not well characterised. However, we recently showed that the sRNAs generated from these regions are exported in *H. bakeri* EVs and are produced by RdRPs, contain a 5′ triphosphate, and start with a G. These are specifically associated with an extracellular Worm-specific Argonaute protein (exWAGO) which is packaged in EVs (Chow et al., 2019). Our work here suggests the sRNAs in *T. muris* EVs do not contain a 5′ triphosphate and we did not identify a close ortholog to exWAGO in *T. muris*. It is possible the sRNAs mapping to intergenic and repetitive regions represent another class of siRNA, for which we do not yet know the mechanism of biogenesis or export in EVs. It seems unlikely that the *T. muris* sRNAs from repetitive or intergenic regions are simply degradation products since they show some size preference to be ~21–25 nt in length, unlike ribosomal degradation products which are more variable in length (Supplementary Fig. S3). There is emerging data suggesting sRNAs from repetitive regions and transposable elements can in fact act as virulence factors in diverse parasite-host models including plant-parasite interactions (Cai et al., 2019, Dunker et al., 2020, Hudzik et al., 2020). These classes of extracellular sRNAs that are found across diverse nematodes and other helminths therefore merit further attention.

To date the vast majority of research on extracellular sRNAs has focused on miRNAs, a very well characterised type of sRNA that has a conserved biogenesis pathway in animals. We recently showed that parasite miRNAs could be detected in macrophages in the pleural cavities of mice infected with the filarial nematode *L. sigmodontis* (Quintana et al., 2019) and others have shown that miRNAs from *S. japonicum* are detected in macrophages *in vivo*, and incorporate into mouse Argonaute 2 *in vitro* (Liu et al., 2019)*.* Our work here underscores the reliability of miRNAs detected across different datasets, and laboratories *in vitro*, and revealed a very distinct composition of parasite miRNAs in EVs from *H. bakeri* versus *T. muris*. It is hypothesised that the EV sRNA cargo would enable specific targets and functions in the host which could contribute to modulating the distinct niches in which these parasites reside. For example, the duodenum of the small intestine and the caecum differ in many ways, including their architecture, the small intestine contains villi while the caecum does not, as well as differing in the proportion of certain specialised epithelial cell types, with Paneth cells only residing in the small intestine, and goblet cells increasing along the length of the intestine (Mowat and Agace, 2014). In an accompanying paper we show that *T. muris* EVs causes a suppression of type I interferon response in caecaloids (Duque-Correa et al., 2020) and an exciting future direction will be comparing the uptake and function of EVs from *H. bakeri* and *T. muris* on enteroids versus caecaoloids in order to further assess the role of the sRNA cargo in mediating different host gene expression changes induced by each parasite. Although there is still a lack of quantitative data on extracellular sRNAs (how much must be imported for gene regulation in the recipient cell) (Claycomb et al., 2017), increasing evidence in many mammalian disease contexts shows functional effects of extracellular miRNAs using genetic tools (Fritz et al., 2016, Mateescu et al., 2017). We previously showed that miRNAs from *H. bakeri* EVs could be transmitted into host cells via EVs and used a reporter assay to demonstrate the synthetic nematode miRNAs can directly bind to, and suppress, host genes including the phosphatase Dusp1 (Buck et al., 2014) which is associated with cytokine regulation in helminth infection (Klotz et al., 2011). A study comparing EV cargo from different *Ascaris suum* larval stages showed some miRNAs were distinct to the EVs from L3s compared with those from other larval stages (Hansen et al., 2019). Intriguingly, *A. suum* L3s migrate through a number of host tissues, and it has been suggested that L3-specific miRNA cargo could target host processes such as tissue lysis, repair and/or innate immunity (Hansen et al., 2019). At present it remains a challenge to accurately identify and validate the host targets of parasite miRNAs, since each parasite miRNA will be predicted to target hundreds to thousands of host genes and many of these predictions could be false positives. Furthermore, it may not be appropriate to assume seed-based pairing is the only or main criteria for recognition, as is proposed to occur for endogenous miRNAs (Bartel, 2018). Finally, we cannot rule out interactions between parasite sRNAs including miRNAs and other co-inhabiting organisms (for example microbes or other parasites) (Liu et al., 2016). Whilst daunting to address all of these aspects, biochemical methods to directly identify sRNA-target interactions have advanced substantially in the last 5 years (Moore et al., 2015) and the application of these methods to helminth studies could significantly advance the field. Given the beneficial aspects of helminths in auto-immune and allergic contexts, it will be exciting to determine the sRNA molecules that may be contributing to immune suppression and modulation of the intestinal epithelial cell niche (Duque-Correa et al., 2020). Furthermore, the study of these natural parasite EVs may help teach us how to safely and specifically deliver foreign or synthetic sRNA to cells without eliciting an immune response, which is urgently needed in the field of RNA therapeutics.
