# Supplementary material for: Extracellular vesicles from Heligmosomoides bakeri and Trichuris muris contain distinct microRNA families and small RNAs that could underpin different functions in the host
Source: Int J Parasitol. 2020 Aug;50(9):719–29. doi: 10.1016/j.ijpara.2020.06.002 (PMC7435682; doi:10.1016/j.ijpara.2020.06.002)

Supplemental Fig. S1.

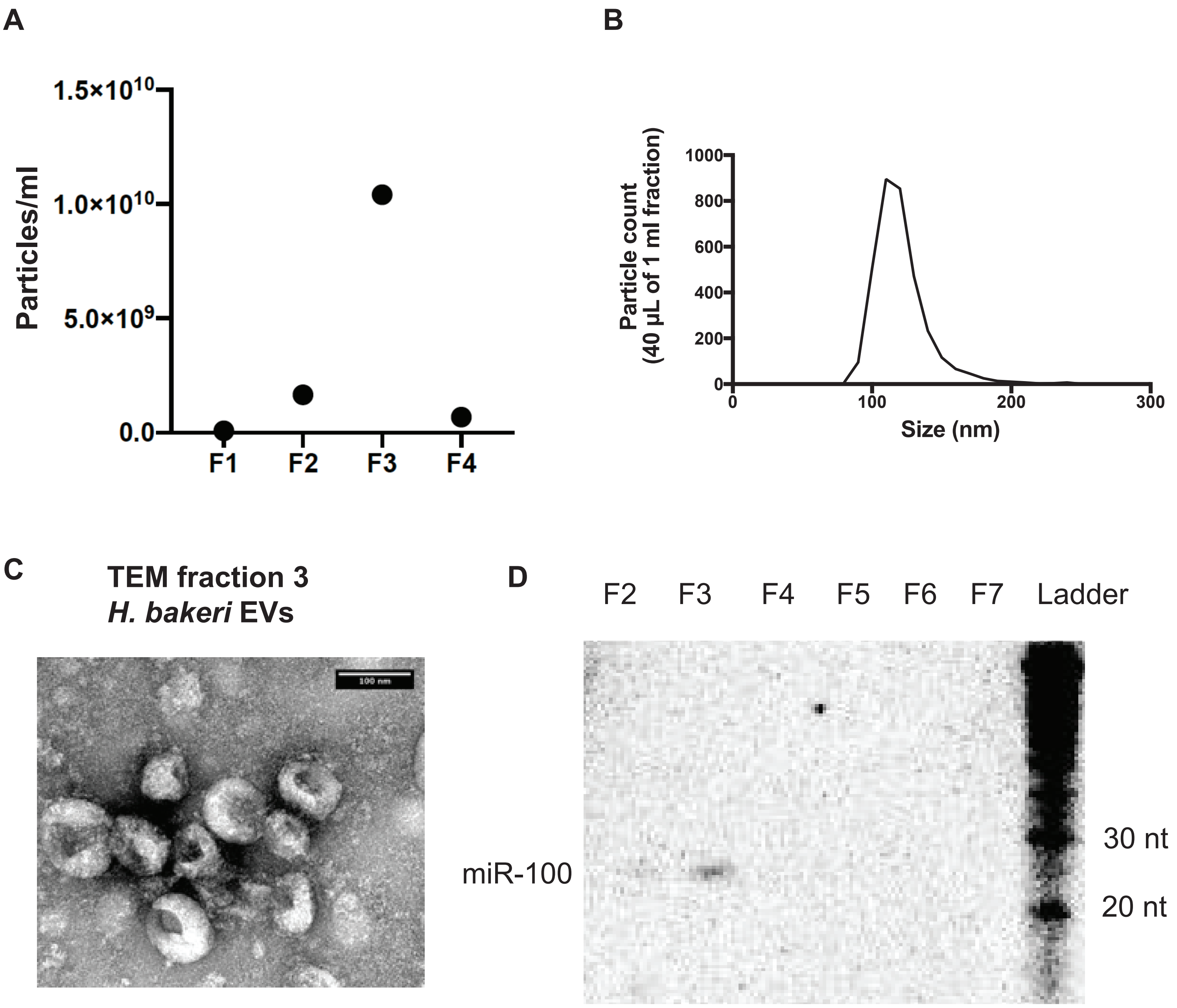

Supplemental Fig. S2.

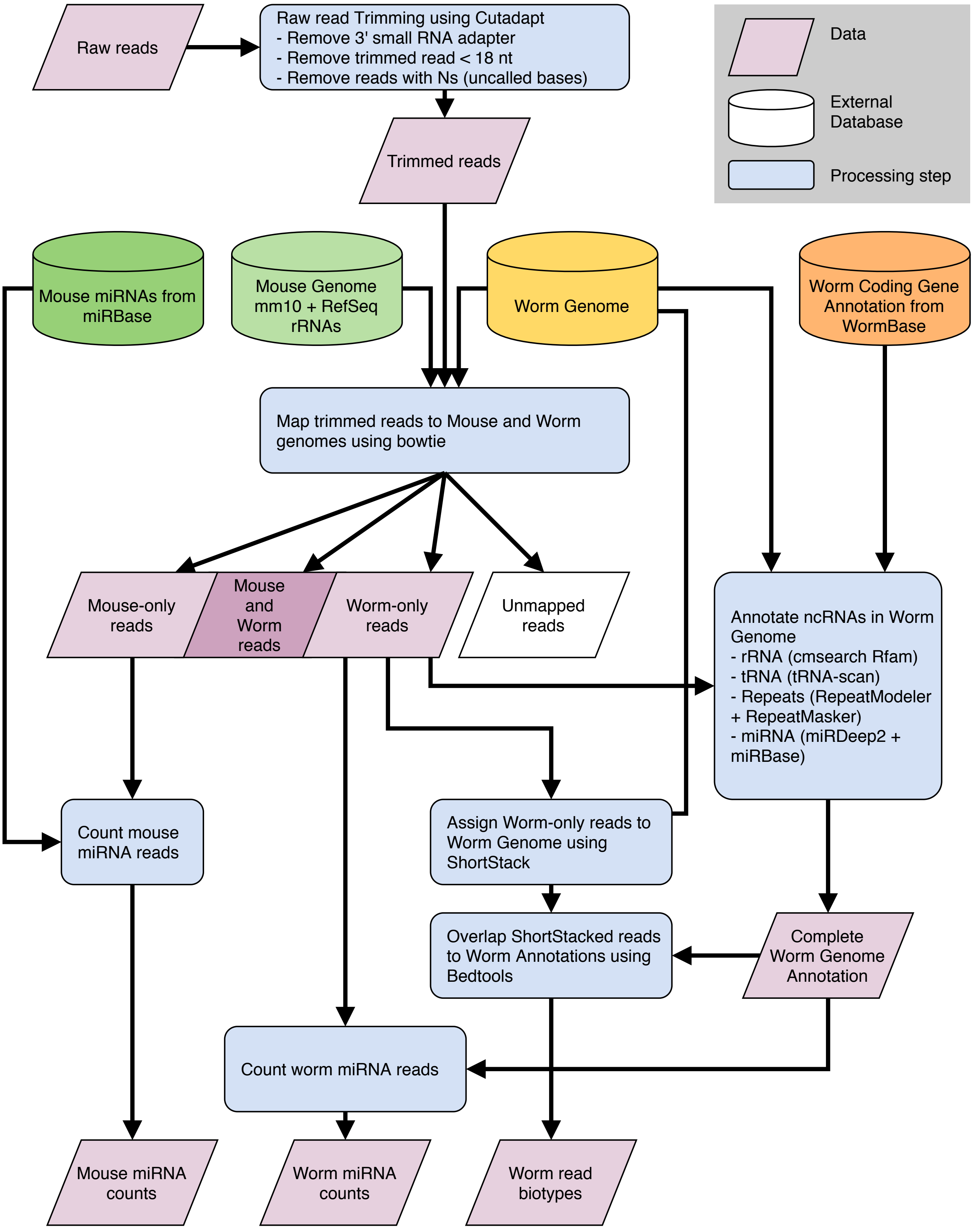

Supplemental Fig. S3.

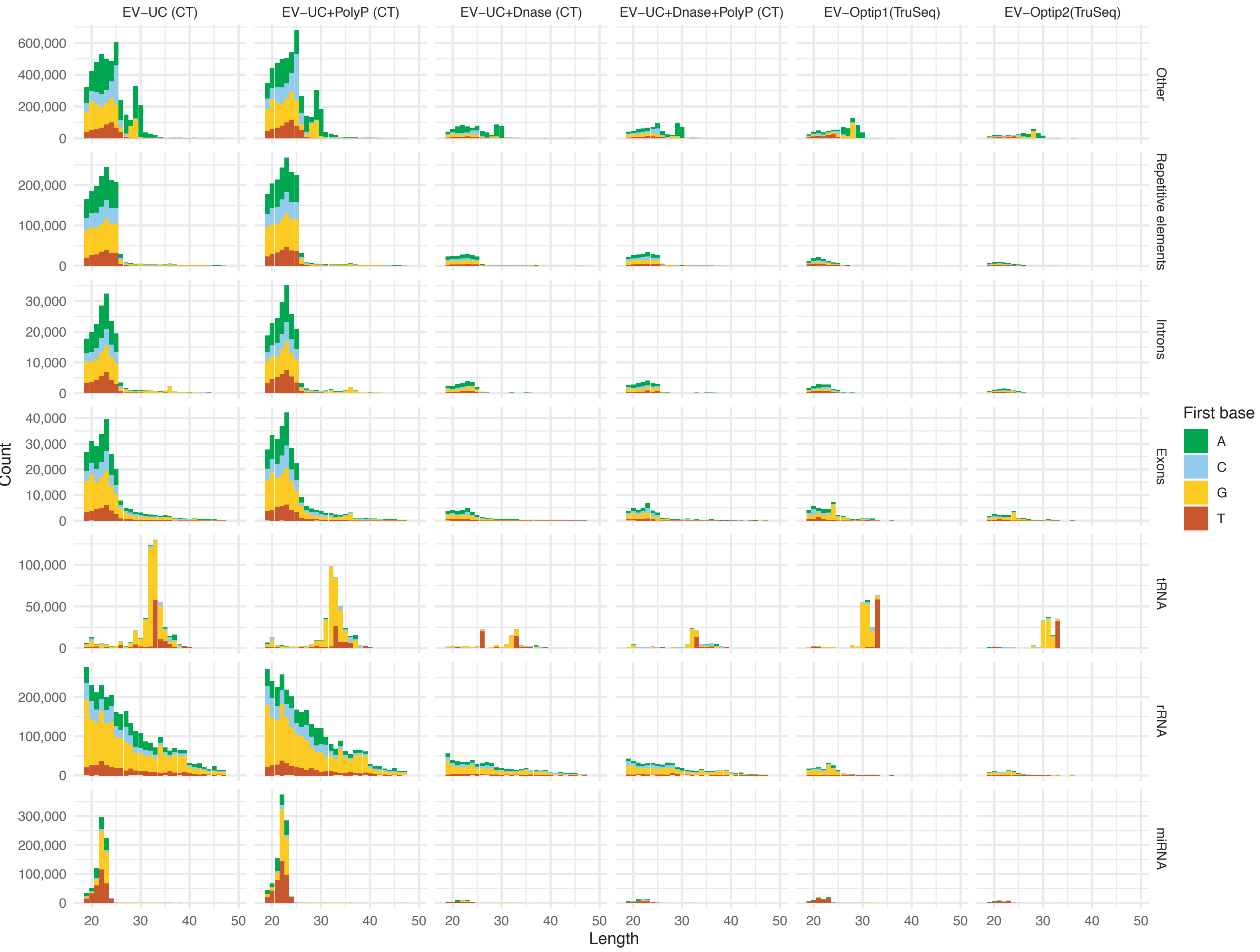

Supplemental Fig. S4.

A

H. bakeri EV libraries

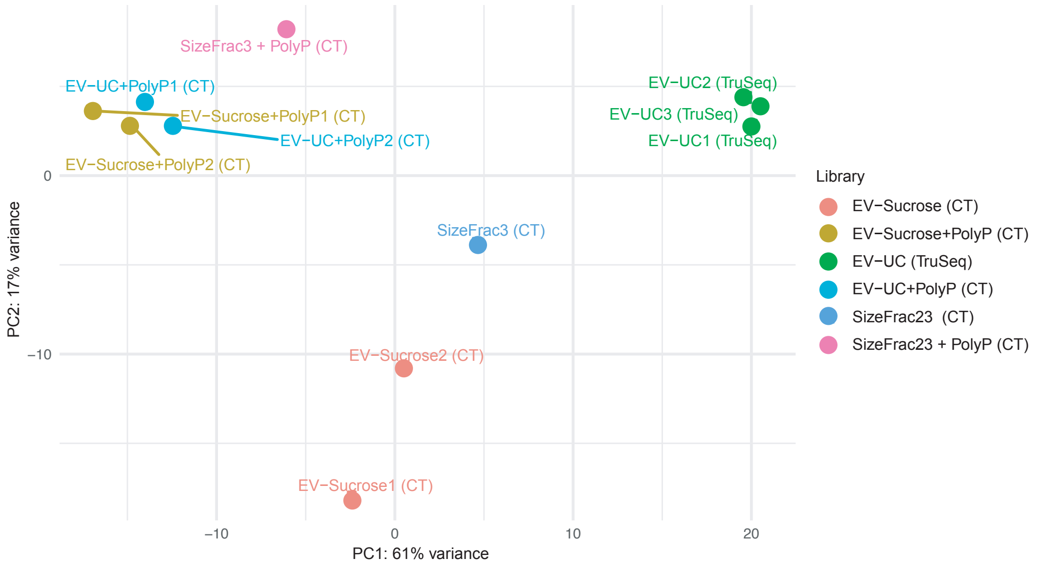

B

T. muris EV libraries

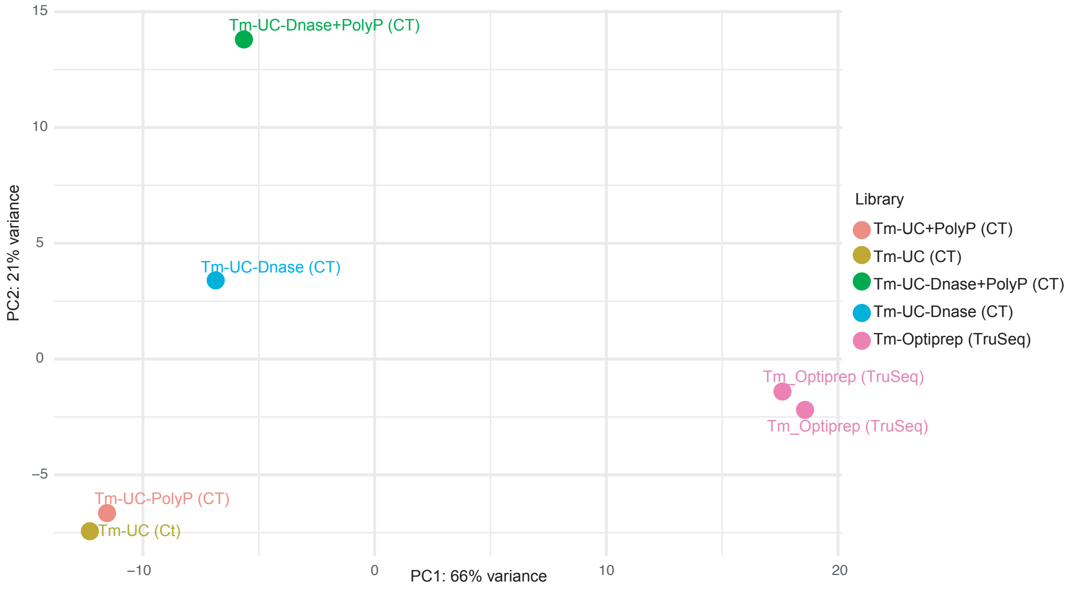

Supplemental Fig. S5.

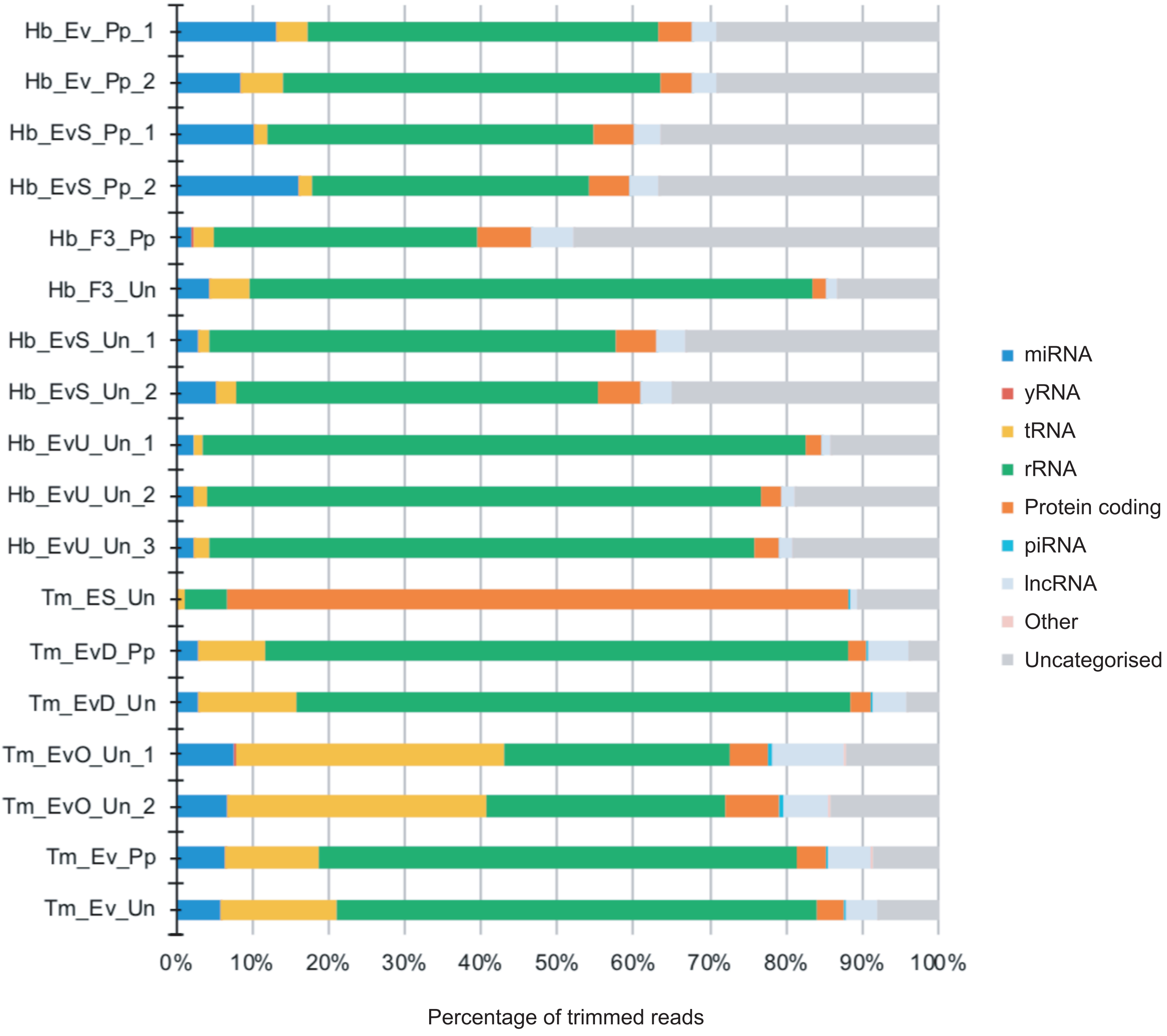

Supplemental Fig. S6.

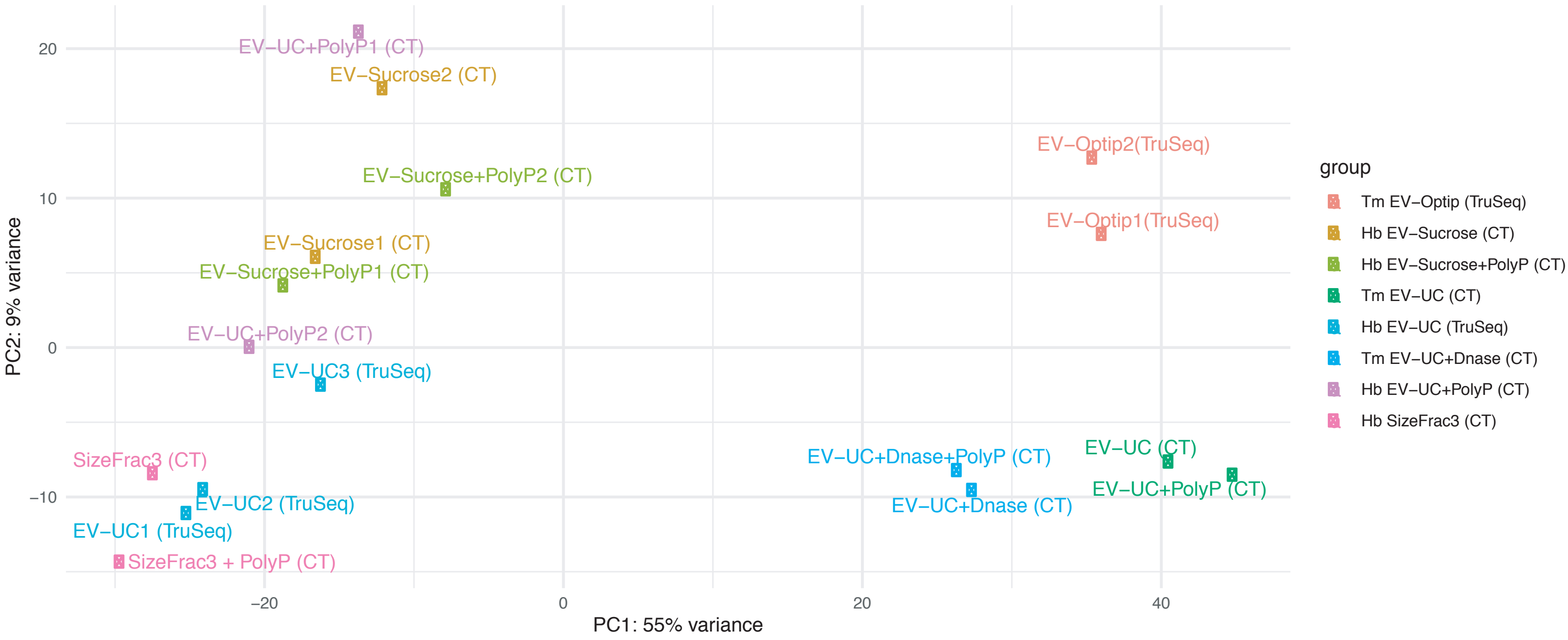

Supplement: Supplementary data 1 [file mmc1.pdf]
